# TPC1 Has Two Variant Isoforms, and Their Removal Has Different Effects on Endo-Lysosomal Functions Compared to Loss of TPC2

**DOI:** 10.1128/MCB.00113-14

**Published:** 2014-11

**Authors:** Margarida Ruas, Kai-Ting Chuang, Lianne C. Davis, Areej Al-Douri, Patricia W. Tynan, Ruth Tunn, Lydia Teboul, Antony Galione, John Parrington

**Affiliations:** a Department of Pharmacology, University of Oxford, Oxford, United Kingdom; b The Mary Lyon Centre, MRC Harwell, Harwell, United Kingdom

## Abstract

Organelle ion homeostasis within the endo-lysosomal system is critical for physiological functions. Two-pore channels (TPCs) are cation channels that reside in endo-lysosomal organelles, and overexpression results in endo-lysosomal trafficking defects. However, the impact of a lack of TPC expression on endo-lysosomal trafficking is unknown. Here, we characterize *Tpcn1* expression in two transgenic mouse lines (*Tpcn1*^XG716^ and *Tpcn1*^T159^) and show expression of a novel evolutionarily conserved *Tpcn1B* transcript from an alternative promoter, raising important questions regarding the status of *Tpcn1* expression in mice recently described to be *Tpcn1* knockouts. We show that the transgenic *Tpcn1*^T159^ line lacks expression of both *Tpcn1* isoforms in all tissues analyzed. Using mouse embryonic fibroblasts (MEFs) from *Tpcn1*^−/−^ and *Tpcn2*^−/−^ animals, we show that a lack of *Tpcn1* or *Tpcn2* expression has no significant impact on resting endo-lysosomal pH or morphology. However, differential effects in endo-lysosomal function were observed upon the loss of *Tpcn1* or *Tpcn2* expression; thus, while *Tpcn1*^−/−^ MEFs have impaired trafficking of cholera toxin from the plasma membrane to the Golgi apparatus, *Tpcn2*^−/−^ MEFs show slower kinetics of ligand-induced platelet-derived growth factor receptor β (PDGFRβ) degradation, which is dependent on trafficking to lysosomes. Our findings indicate that TPC1 and TPC2 have important but distinct roles in the endo-lysosomal pathway.

## INTRODUCTION

The endo-lysosomal system plays an important role in a multiplicity of cellular processes, including endocytotic and secretory events and protein/organellar degradation. As such, it is involved in processes as varied as growth and differentiation (1), synaptic transmission (2), immune functions (3), autophagy (4), and microbial pathogenicity (5), to name but a few. Physiological functions of the endo-lysosomal system are dependent on ion homeostasis, which in turn regulates organelle acidification, fusion and fission events, and enzymatic activity (6). It is therefore not surprising that the activities of the ion channels, pumps, and transporters present within the membranes of specific endo-lysosomal organelles have an impact on the overall function of this system, with mutations in these proteins being associated with human disease (7, 8).

Recently, a new family of endo-lysosomal cation channels, the two-pore channel (TPC) proteins, has been identified (9–12). TPCs are predicted to have 12 transmembrane helices distributed over two domains and are considered intermediates between the single-domain cation channels, such as CatSpers and transient receptor potential (TRP) channels, and the four-domain α subunits of the voltage-gated Ca^2+^ channels (VGCCs) (13, 14). In animals, TPC proteins are encoded by three different genes (gene nomenclature, *TPCN1*, *TPCN2*, and *TPCN3*), with *TPCN3* being absent in some species, including humans and mice (15). Although TPC proteins localize within the endo-lysosomal system, each of the TPC isoforms shows a somewhat different organellar distribution, as assessed by heterologous expression systems. Thus, while TPC1 seems to have a broad pattern of colocalization with markers for recycling endosomes, early and late endosomes, and lysosomes (10, 12, 16), TPC2 predominantly colocalizes with markers for late endosomes and lysosomes (10, 11, 16), with mutations in an N-terminal lysosomal targeting dileucine motif (17) present in human TPC2 resulting in mistargeting of the mutant protein to the plasma membrane (18).

Over the last few years, numerous studies have investigated the mode of regulation of TPC activity, with several lines of evidence indicating that TPCs function as Ca^2+^-release channels gated by the intracellular messenger nicotinic acid adenine dinucleotide phosphate (NAADP) (19), a chemical messenger that targets the release of Ca^2+^ from acidic, endo-lysosomal organelles (20). Furthermore, mutations affecting the *N*-glycosylation status of TPC1 potentiate NAADP action (21), and other factors, such as pH (22–26), voltage (24, 25), Ca^2+^ (22, 24), Mg^2+^ (27), and kinase activity (27, 28), have been shown to influence the properties of mammalian TPCs, with differences being observed between TPC1 and TPC2. Additionally, recent reports have indicated that TPCs can also conduct Na^+^ and H^+^ and be regulated by the phosphoinoside lipid phosphatidylinositol 3,5-bisphosphate [PI(3,5)P_2_] (26–29), raising the possibility that these channels have a cation permeability broader than those that have been considered until now and modes of regulation in addition to those that have been considered.

In contrast to what is known about the regulation of TPC activity by factors such as the ones mentioned above, little information is available about factors regulating TPCs at the level of gene expression. Such information could lead to a better understanding of the mechanisms responsible for the differences in the levels of the transcripts of the different TPC isoforms that have been observed in different tissues (9–11), throughout embryonic development (30), and throughout cellular differentiation (30–33). Differences in the levels of expression could potentially have an impact on physiological processes where TPC action has been implicated. Our previous studies have indicated that overexpression of TPCs in a cell line causes impairment in the endo-lysosomal system, with cells acquiring morphological abnormalities similar to those observed in endo-lysosomal storage disorders and impairment in trafficking (16); other recent studies have suggested a role for TPC2 in autophagy (31, 34). These findings could indicate a potential role for abnormalities of TPC expression in disorders of the endo-lysosomal system. However, the impact of the loss of the TPC1 or TPC2 protein upon endo-lysosomal trafficking still remains to be investigated.

In this study, we report the existence of a novel, alternative variant isoform of *Tpcn1*, termed *Tpcn1B*, resulting from an alternative promoter and giving rise to a TPC1B protein with an N-terminal truncation relative to the sequence of the TPC1A isoform previously described (9, 10, 12). We have developed mice deficient for expression of both *Tpcn1* isoforms, and studying mouse embryonic fibroblasts (MEFs) derived from these *Tpcn1*^−/−^ animals or *Tpcn2*^−/−^ mice previously developed (10), we observed differential effects on endo-lysosomal trafficking following the loss of each isoform.

## MATERIALS AND METHODS

### Animals.

*Tpcn1*^XG716^ mice [transgenic allele nomenclature, *Tpcn1*^Gt(XG716)Byg^] were generated from the ES cell line XG716 (Bay Genomics). This line carries a gene trap cassette from the pGT1Lxf vector in a *Tpcn1* allele in the intron between exons 2 and 3. Cells from this ES cell line (129P2/OlaHsd background) were injected into C57BL/6 mouse blastocysts to produce chimeric mice that were bred with further C57BL/6 mice to obtain germ line transmission of the *Tpcn1*^XG716^ allele. Animals homozygous for wild-type (WT) and *Tpcn1*^XG716^ alleles were obtained from heterozygote crosses, and separate colonies were maintained. *Tpcn1*^T159^ mice (transgenic allele nomenclature, *Tpcn1*^tm1Dgen^) carry a targeted disruption of exons 4 and 5 and were obtained in a C57BL/6;129P2 background from the European Mouse Mutant Archive (EMMA). WT and *Tpcn1*^T159^ colonies were generated as described above. *Tpcn2*^−/−^ mice [*Tpcn2*^YHD437^; transgenic allele nomenclature, *Tpcn2*^Gt(YHD437)Byg^] were described previously (10). Mice were housed in individual ventilated cages with a constant optimal temperature and humidity and with 12 h of light per day in the Biomedical Science Building (Oxford University). Mice were fed standard dry pellets and were provided water *ad libitum*. Animal use was approved by the University of Oxford's Local Ethical Review Committee and was permitted by a license from the United Kingdom Home Office in accordance with United Kingdom law (the Animals [Scientific Procedures] Act 1986).

### Genotyping.

For determination of the gene trap insertion site in the *Tpcn1*^XG716^ allele, genomic DNA was extracted from ES cells carrying that allele following a standard procedure using phenol-chloroform-isoamyl alcohol. PCRs were performed using an Expand Long Range PCR system (Roche) and the primers and conditions described in Table S1 in the supplemental material, and the products were analyzed by agarose gel electrophoresis. The PCR products were cloned into pCRIITOPO (Invitrogen) and sequenced for determination of the insertion site. Subsequently, DNA from mouse ear clips was extracted by digestion overnight at 55°C in lysis buffer (50 mM Tris, 5 mM EDTA, 0.1% SDS, pH 8.0, 1 mg/ml proteinase K), followed by proteinase K inactivation at 95°C for 15 min. The genotyping of animals was performed using a 3-primer PCR protocol and the primers and conditions described in Table S2 in the supplemental material.

### MEFs.

MEFs were prepared from embryonic day 13. 5 embryos using standard protocols (35) and either used as primary cells at low passage (<5 passages, for trafficking studies) or immortalized by serial passaging (for TPC localization studies). Cells were maintained at 37°C in 5% CO_2_ in growth medium composed of Dulbecco modified Eagle medium (DMEM), 10% fetal calf serum, 100 U/ml penicillin, 100 μg/ml streptomycin, and 2 mM glutamine.

### Short hairpin RNA (shRNA) knockdown.

Lentiviral pLKO.1 constructs carrying hairpin sequences against mouse *Rhob* (clone TRCN0000077537) and mouse *Rab7b* (clone TRCN0000100657) transcripts were obtained via Open Biosystems. A control pLKO.1 scrambled construct (clone 1864 [36]) was obtained from Addgene. Lentiviruses were produced by jetPEI transfection reagent-mediated (Source Biosciences) cotransfection of LentiX-293T cells (Clontech) with vesicular stomatitis virus G glycoprotein (VSV-G) envelope plasmid (pMD2.VSVG), packaging plasmid (pCMV ΔR8.91), and pLKO.1 constructs. The transfection medium was changed after an overnight incubation and conditioned twice for 24 h. The conditioned media were pooled and filtered through a 0.45-μm-pore-size filter, and aliquots were stored at −80°C until use. For transduction, lentivirus-conditioned medium containing 8 μg/ml Polybrene was added to WT MEFs, followed by centrifugation at 1,500 × *g* for 1 h. Sixteen to 24 h later the medium was changed, and the cells were analyzed the day after.

### RT-PCR and RT-qPCR analysis.

Harvested mouse tissues were immediately frozen in liquid nitrogen and stored at −80°C until use. Frozen mouse tissue was lysed and homogenized using an Ultra-Turrax homogenizer. Total RNA was extracted using an RNeasy QiaRNA extraction procedure (Qiagen) with an in-column DNase I treatment. The eluted RNA was stored at −80°C. The integrity of the RNA was assessed by agarose gel electrophoresis. Reverse transcription (RT)-PCR was performed in a reaction mixture containing extracted total RNA, SuperScript III reverse transcriptase/Platinum *Taq* high-fidelity enzyme mix (Invitrogen), and gene-specific primers. The primers and reaction conditions are described in Table S3 in the supplemental material. The reaction products were analyzed by agarose gel electrophoresis. The identity of the products was confirmed by sequencing gel-extracted PCR products for each set of primers.

For RT-quantitative PCR (qPCR), cDNA was synthesized from RNA (prepared as described above) using a high-capacity cDNA reverse transcription kit (Applied Biosystems). cDNA was subjected to qPCR using gene-specific primers and corresponding universal probes in a LightCycler 480 system (Roche). The primers and qPCR probes are described in Table S4 in the supplemental material. For *Tpcn1*, copy numbers were determined against a standard curve using recombinant RNA sequences corresponding to each amplicon as the template for the initial cDNA synthesis, and the copy number per ng RNA was determined by normalization to the copy numbers of three genes, *Gapdh* (for glyceraldehyde-3-phosphate dehydrogenase), *Cyc1*, and *Actb*. For *Rhob* and *Rab7b*, relative mRNA levels were determined by normalization to the levels of *Gapdh*, *Cyc1*, and *Rn18s* (18S rRNA).

### Tissue homogenates.

Tissues from individual animals were collected and washed in phosphate-buffered saline (PBS). They were then suspended in homogenization buffer (20 mM HEPES, 1 mM EDTA, pH 7.2) containing protease inhibitors (Complete; Roche) and homogenized using an Ultra-Turrax homogenizer. The homogenate was cleared of undisrupted cells and nuclei by centrifugation at 1,000 × *g* for 5 min at 4°C. The supernatant was then centrifuged at 100,000 × *g* for 1 h at 4°C, and the membrane pellet was resuspended in homogenization buffer containing protease inhibitors. Aliquots were stored at −80°C. Protein content was determined using a bicinchoninic acid (BCA) assay.

### Immunoblotting analysis.

Protein samples were mixed in Laemmli sample buffer containing 200 mM dithiothreitol (heat treatment was omitted for detection of TPC1 proteins). Samples were resolved by SDS-PAGE and transferred to polyvinylidene difluoride membranes. Antibody incubations were performed with affinity-purified anti-TPC1 polyclonal rabbit antibodies custom-made using immunogenic human TPC1 peptides (IQEWYEEHAREQEQQR and APAAQQPPGSRQRSQTVT), anti-β-galactosidase mouse monoclonal antibody (clone 40-1a; Developmental Studies Hybridoma Bank), or antimultired rat monoclonal antibody (clone 5F8, provided by Heinrich Leonhardt [37]). After incubation with secondary horseradish peroxidase-conjugated antibodies, membranes were developed by chemiluminescence (ECL Advance; GE Healthcare).

### Colocalization studies.

Mouse *Tpcn1A* and *Tpcn2* cDNAs were obtained from Source BioScience (Image clones 6821376 and 9055807, respectively). *Tpcn1B* cDNA was obtained by RT-PCR from mouse liver RNA (using the primers described in Table S3 in the supplemental material), and the resulting products were cloned into pCRIITOPO (Invitrogen). cDNAs were then amplified by PCR using primers to introduce restriction sites for ligation into a pcDNA5TO vector (Invitrogen) with a C-terminal mCherry sequence, and the resulting constructs were sequenced. For transfections, immortalized MEFs were grown on poly-d-lysine-treated coverslips and cotransfected using the JetPEI reagent (Polyplus transfections) with TPC with a C-terminal mCherry tag (TPC.mCherry) constructs and green fluorescent protein (GFP)-tagged markers for early endosomes (EEA1, provided by Silvia Corvera), recycling endosomes (TfR, provided by Philip Woodman), late endosomes/lysosomes (Lamp1, provided by Esteban C. Dell'Angelica), or endoplasmic reticulum (ER; KDEL, provided by Sergio Grinstein). After 48 h, cells were fixed with 4% paraformaldehyde in PBS and mounted on slides with ProLong Gold antifade reagent (Invitrogen). Cells were viewed on a Zeiss 510 Meta confocal microscope in multitrack mode, using the following excitation/emission wavelengths (nm): for GFP, 488/505 to 530; for mCherry 543/>560. Pearson coefficients were calculated for individual cells using Zeiss LSM software.

### Growth curves.

Primary MEFs were plated on 12-well plates at 4 × 10^4^ cells/well, and the medium was changed every 2 days. For each time point, cells were washed in PBS, fixed for 15 min in 4% paraformaldehyde, and washed in water. Cells were then stained with 0.1% crystal violet for 30 min and washed four times with water. Retained dye was eluted in 10% acetic acid, and the absorbance at 590 nm was measured. The results were normalized to the readings obtained 1 day after plating. Data from 2 different experiments performed in triplicate were fitted to an exponential growth curve, and doubling times were determined (GraphPad Prism).

### Endo-lysosomal pH.

Primary MEFs were loaded by endocytosis with fluorescein-dextran (pH sensitive) and Texas Red-dextran (pH insensitive) at 0.2 mg/ml in growth medium in 96-well plates at 37°C for 16 h. The cells were washed three times with dextran-free medium and immediately processed. Fluorescence measurements were collected from labeled endo-lysosomes using a Novostar plate reader (BMG Labtech) using excitation/emission of 485/520 nm for fluorescein and 570/620 nm for Texas Red. Autofluorescence (cells not loaded with dextrans) was subtracted from the fluorescein and Texas Red fluorescence. Fluorescein fluorescence (*G*) was divided by the Texas Red fluorescence (*R*), and the pH was determined against a standard curve obtained in the following way: dextran-loaded MEFs were equilibrated for at least 10 min in a high-K^+^ extracellular buffer (5 mM NaCl, 145 mM KCl, 1 mM MgCl_2_, 1 mM CaCl_2_, 10 mM glucose) and adjusted to a series of defined pH values in buffers (10 mM acetate for pH 4 to 5, 10 mM MES [morpholineethanesulfonic acid] for pH 5.5 to 6.5, and 10 mM HEPES for pH 7) containing 2 μM nigericin and 2 μM valinomycin. Fluorescence measurements were acquired as described above. Fluorescein- and Texas Red-dextrans (molecular weight, 10,000) were purchased from Invitrogen.

### Electron microscopy.

Pellets of confluent primary MEFs were fixed on ice for 15 min in the presence of 0.1% glutaraldehyde (from TAAB Laboratories Equipment). The solution was carefully removed and replaced by layering a 0.1 M cacodylate buffer solution (pH 7.4) containing 2.5% glutaraldehyde and 20 mM CaCl_2_. Dislodged pellets were further fixed for 1 h at room temperature. After washing in 0.1 M cacodylate buffer solution and postfixing in 1% osmium tetroxide for 1 h at room temperature, the pellets were washed again, rinsed with 70% ethanol, and then block stained with 1% uranyl acetate in 70% ethanol. The pellets were serially dehydrated using ice-cold solutions of 80%, 90%, 95%, and 100% ethanol. After 3 washes with 100% ethanol at room temperature, the pellets were further washed twice with propylene oxide. The pellets were infiltrated with resin-propylene oxide (1:1) for 1.5 h, and the propylene oxide was allowed to evaporate for 45 min. Pellet fragments were embedded in plastic and polymerized for 3 days at 60°C. All the fixation and infiltration steps were carried out on a shaking platform. Finally, 1-μm sections of resin blocks were stained with a 1% toluidine blue solution in 1% borax. Selected areas were cut at 50 to 70 nm, and the sections were collected on Pioloform polyvinyl butyral-coated single-slot copper grids. The sections were stained on the grid for 2 to 5 min with Reynold's lead solution to enhance contrast in the electron microscope. The photographic plates were taken on a Leica transmission electron microscope.

### Cholera toxin uptake.

Primary MEFs grown on poly-d-lysine-treated coverslips were incubated on ice for 30 min in Hanks balanced salt solution buffer (Sigma) containing 1 μg/ml Alexa Fluor 555-labeled subunit B of cholera toxin (CTxB; Invitrogen). After unbound CTxB was washed, growth medium was added and the cells were incubated at 37°C for the times indicated below. After the chase period, cells were fixed with 4% paraformaldehyde in PBS and permeabilized with 0.1% Triton X-100. Immunolabeling of the Golgi apparatus was performed with an anti-GM130 mouse monoclonal antibody (35/GM130; BD Biosciences) and an Alexa Fluor 488-labeled anti-mouse IgG (Invitrogen). Coverslips were mounted on slides with ProLong Gold antifade reagent with DAPI (4′,6-diamidino-2-phenylindole; Invitrogen). Cells were imaged by confocal microscopy with appropriate excitation/emission settings for detection of Alexa Fluor 555, Alexa Fluor 488, and DAPI. The proportion of CTxB localized in the Golgi apparatus was calculated by producing binary layers by thresholding the CTxB and Golgi apparatus channels. Threshold parameters were kept constant for all cells analyzed, and the proportion of total CTxB colocalizing with the Golgi apparatus was calculated.

### PDGFRβ degradation.

Primary MEFs grown on 96-well plates were serum starved for 24 h in DMEM containing 0.2% bovine serum albumin, 100 U/ml penicillin, 100 μg/ml streptomycin, and 2 mM glutamine. Cells were then pretreated for at least 30 min with 20 μg/ml of cycloheximide, before addition of 20 ng/ml platelet-derived growth factor BB (PDGF-BB; Gibco). At the time points indicated below, cells were washed in ice-cold PBS, fixed in 4% paraformaldehyde in PBS, and permeabilized with 0.1% Triton X-100. Immunofluorescence was performed using an anti-platelet-derived growth factor receptor β (anti-PDGFRβ) antibody (28E1; Cell Signaling Technology) and an IRDye 800-labeled secondary antibody (LI-COR). Relative cell numbers were measured using CellTag 700 stain (LI-COR). Fluorescence signals were detected on an Odyssey infrared scanner (LI-COR) using an in-cell Western acquisition mode. The results were normalized to the readings obtained for equivalent PDGF-BB-untreated samples. Data were fitted to a one-phase decay curve, and half-lives were determined (GraphPad Prism).

### Bioinformatics.

Exon numbering for *Tpcn1* is based on the genomic sequence of the gene with identification (ID) no. 252972 and the cDNA sequence with GenBank accession number NM_145853.2, referred to in this study as *Tpcn1A*. The variant transcript arising from an alternative promoter described in this study is referred to as *Tpcn1B*. Promoter analysis was performed on the basis of data from cap analysis of gene expression (CAGE; accessed via http://fantom3.gsc.riken.jp/) and the Encyclopedia of DNA Elements (ENCODE; accessed via http://genome.ucsc.edu/ENCODE/).

## RESULTS AND DISCUSSION

### Expression of *Tpcn1* is not abolished in *Tpcn1*^XG716^ mice.

**FIG 1:**
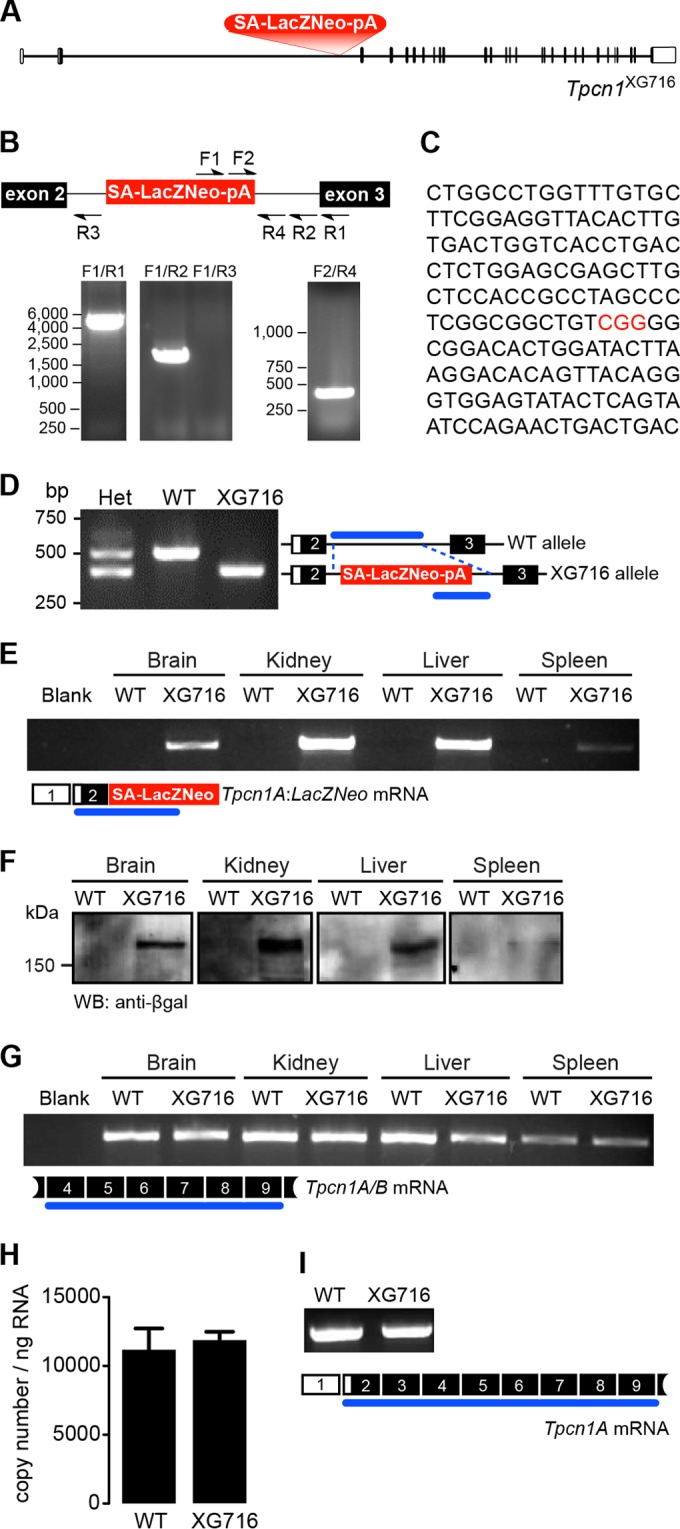
The gene trap mutation in *Tpcn1*^XG716^ does not abolish *Tpcn1* expression. (A) Schematic representation of *Tpcn1*^XG716^ gene structure based on the gene with GenBank accession number NM_145853.2. Red block, gene trap sequences (SA, splice acceptor; LacZNeo, chimeric β-galactosidase and neomycin phosphotransferase II sequences; pA, polyadenylation signal); vertical segments, exons; unfilled sections, UTRs. (B) PCR strategy for determination of the insertion site of gene trap vector pGT1Lxf in the *Tpcn1*^XG716^ allele. Several combinations of forward (F) and reverse (R) primers were used to narrow the location of the gene trap vector within the intron. (C) The product from PCR with primers F2/R4 was cloned into pCRIITOPO (Invitrogen) and sequenced, and the insertion site of the gene trap within the intron was determined to be between 2,754 and 2,757 bp upstream of exon 3 (marked in red). (D) Genotyping for WT and *Tpcn1*^XG716^ alleles. Numbered white and black blocks, noncoding and coding exons, respectively; blue lines, PCR-amplified regions; Het, heterozygote. (E) RT-PCR analysis of the expression of the chimeric trapped *Tpcn1*^XG716^ transcript (*Tpcn1A-LacZ-Neo*) from WT and homozygote *Tpcn1*^XG716^ animal tissues. Blank, a reaction with no RNA. (F) Immunoblotting analysis of chimeric TPC1A–β-galactosidase–neomycin phosphotransferase II protein in tissue homogenates from WT and homozygote *Tpcn1*^XG716^ animals using an anti-β-galactosidase (anti-β-gal) antibody. WB, Western blotting. (G) Expression of *Tpcn1A/B* determined by probing for transcript regions downstream from the gene trap insertion in tissues from WT and homozygote *Tpcn1*^XG716^ animals. (H) Copy number of *Tpcn1A/B* transcripts in liver from WT and homozygote *Tpcn1*^XG716^ animals determined by RT-qPCR. Bars correspond to the mean ± SEM for 4 to 8 animals. (I) Expression of the *Tpcn1A* transcript in the liver resulting from skipping of the gene trap cassette.

We have previously developed and characterized a mouse line deficient for *Tpcn2* expression that we used to show the requirement for the TPC2 protein in NAADP-evoked cation currents in pancreatic β-cells (10) and in contractile responses to NAADP in detrusor smooth muscle (38). To extend our studies to encompass the role of TPC1 in physiological processes, we sought to produce and characterize a *Tpcn1*^−/−^ mouse line. We started by generating a transgenic *Tpcn1* mouse line using the ES cell line XG716; this line carries a gene trap cassette inserted in one *Tpcn1* allele in the intron between exons 2 and 3 (Fig. 1A). Cells from this ES cell line were injected into blastocysts to produce chimeric mice that were bred with further mice to obtain germ line homozygous transmission of the *Tpcn1*^XG716^ allele. After determining the insertion site of the gene trap vector via a series of PCRs and sequencing of the products (Fig. 1B and C), we developed a three-primer genotyping strategy for the simultaneous detection of both wild-type (WT) and *Tpcn1*^XG716^ alleles (Fig. 1D). As predicted, insertion of the gene trap cassette results in the expression of a chimeric mRNA transcript containing sequences from *Tpcn1* exons 1 and 2 joined to the *LacZ-Neo* marker (a fusion of β-galactosidase and neomycin phosphotransferase II sequences) (Fig. 1E) present in the gene trap cassette. The fact that both the chimeric transcript and its corresponding protein are expressed in several tissues from homozygote *Tpcn1*^XG716^ mice (Fig. 1E and F) indicates that the splice acceptor sequence within the gene trap cassette is functional, resulting in a prematurely terminated *Tpcn1* chimeric transcript. Furthermore, this indicates that the levels of β-galactosidase protein and/or activity from cells and tissues derived from these animals could be used as a reporter for *Tpcn1* promoter activity.

RT-PCR probing for *Tpcn1* exonic sequences downstream of the gene trap insertion showed, however, that *Tpcn1* mRNA is still present in *Tpcn1*^XG716^ homozygote mice (Fig. 1G). Further analysis using RT-qPCR on RNA from liver indicated that the copy number of *Tpcn1* RNA observed in *Tpcn1*^XG716^ animals was indistinguishable from that observed in WT animals (Fig. 1H). One of the drawbacks of gene trap mutagenesis employing a splice acceptor within the gene trap cassette, such as the one used to create the *Tpcn1*^XG716^ transgenic line, is the possibility that the cellular splice machinery does not always recognize the inserted splice acceptor sequence, resulting in a mature mRNA identical to the WT form (39). Indeed, RT-PCR analysis using liver RNA from *Tpcn1*^XG716^ homozygotes detected the presence of mRNA sequences encompassing exons 2 (upstream of the gene trap insertion) to 9 (downstream of the gene trap insertion), similar to what was detected in WT animals (Fig. 1I), indicative of posttranscriptional skipping of the gene trap cassette. These results indicate that although the gene trap vector in the *Tpcn1*^XG716^ line is functional, the occurrence of gene trap skipping enables normal *Tpcn1* expression.

### Expression of the variant isoform *Tpcn1B* from an alternative promoter.

**FIG 2:**
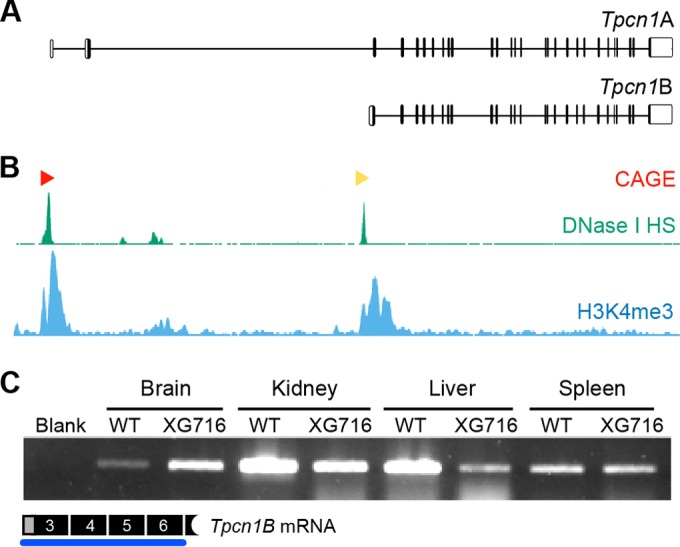
Expression of *Tpcn1B* from an alternative promoter adjacent to exon 3. (A) Schematic representation of genomic organization of *Tpcn1A* and *Tpcn1B* transcript isoforms. Vertical lines, exonic sequences; unfilled boxes, UTRs. Transcription of *Tpcn1B* initiates between exons 2 and 3 at sequences that are intronic relative to *Tpcn1A*. (B) Genomic profiling of markers associated with transcription initiation sites determined by Paraclu clusters of cap analysis of gene expression (CAGE; red and yellow arrowheads, high- and medium-density clusters, respectively, adapted from the FANTOM3 mouse functional annotation database), regions showing DNase I hypersensitivity (DNase I HS; adapted from the UCSC ENCODE/UW data on the basis of results from a transformed mouse mammary adenocarcinoma cell line, the 3134 cell line), and regions with a high incidence of trimethylated lysine 4 of histone 3 (H3K4me3; adapted from the UCSC ENCODE/PSU data on the basis of chromatin immunoprecipitation-sequencing of megakaryocytes). (C) RT-PCR analysis using a *Tpcn1B*-specific forward primer of gene expression from the alternative promoter in tissues from WT and homozygote *Tpcn1*^XG716^ animals.

We next looked at the possibility that transcription of *Tpcn1* initiating at sites downstream from the gene trap cassette in *Tpcn1*^XG716^ adds to the lack of a knockout effect. Northern blot analyses of *Tpcn1* expression in both the rat and mouse have previously revealed the presence of two different-size transcripts (9, 11), with the larger-molecular-size form corresponding to the previously characterized *Tpcn1* isoform (GenBank accession number BC058951; here referred to as *Tpcn1A*). An additional *Tpcn1* cDNA with a molecular weight that roughly corresponds to that of the smaller form revealed in the Northern blots (9, 11) has been detected in cDNA databases (GenBank accession number AK137626) (Fig. 2A); this transcript isoform (here referred to as *Tpcn1B*) contains an alternative 5′ untranslated region (UTR) by initiating from sequences immediately upstream from exon 3 (exon numbering is based on the RefSeq gene with GenBank accession number NM_145853.2) that are intronic relative to *Tpcn1A*. Searches of human cDNA libraries also revealed the presence of equivalent human *TPCN1B* transcripts (GenBank accession numbers AK296268 and AK295022), with several expressed sequence tags (GenBank accession numbers DC341665, DC306526, DC320836, DC307279, DC303859, DC308227, DC307595, DC310669, DC310442, DC323759, DC306238, DC303783, DC310187, DC305810, DC309607, and DC306728) further supporting the expression of the unique exonic sequence of this isoform in humans. These observations seem to indicate that expression of the *Tpcn1B* isoform might be evolutionarily conserved.

We suggest that the expression of the *Tpcn1B* transcript is likely to be driven from an alternative promoter. Indeed, all data available from several genome-wide analyses of parameters known to be associated with transcription regulatory regions—such as (i) cap analysis of gene expression (CAGE), which determines transcription start sites by sequencing the capped 5′ ends of transcripts (40), (ii) regions of DNase I hypersensitivity due to chromatin accessibility (41), and (iii) chromatin immunoprecipitation profiling of the genomic distribution of trimethylated lysine 4 of histone 3, a marker for transcription start sites that correlate with gene activation (41)—point to the presence of an additional promoter adjacent to exon 3 (Fig. 2B). The fact that the two *Tpcn1* isoforms are likely to originate from different promoters and have different 5′ UTRs raises the possibility that their expression is differentially regulated, for example, due to the presence of different transcription factor binding sites and/or different turnover and translation efficiencies of the two mRNA isoforms. These differences could therefore result in distinct relative levels of expression in a tissue- and/or cell-type-specific manner and differential regulation by intracellular signals.

To probe for the expression of *Tpcn1B* in both WT and homozygote *Tpcn1*^XG716^ animals, we performed RT-PCR analysis using a forward primer specific for *Tpcn1B*. Our results showed that this isoform has a broad pattern of tissue expression (Fig. 2C). Furthermore, the gene trap insertion in *Tpcn1*^XG716^ did not abolish the expression of *Tpcn1B*, although different effects were observed among the tissues analyzed (Fig. 2C).

One consequence of the discovery of an alternative promoter driving expression of a novel *Tpcn1B* isoform is the need for a thorough evaluation of *Tpcn1* expression status in transgenic mouse lines recently reported to be *Tpcn1*^−/−^ (28, 29, 42). These studies used different lines of transgenic mice with alterations in the *Tpcn1* gene. One of the lines, *Tpcn1*^LEXKO-471^ [mutant allele nomenclature, *Tpcn1*^Gt(OST359423)Lex^], carries a gene trap insertion in the intron between exons 2 and 3 of *Tpcn1* and was used to provide evidence that the loss of TPC1 expression does not affect binding of a cross-linkable NAADP-based probe (42). On the basis of information available for the *Tpcn1*^LEXKO-471^ transgenic line (accessed via the European Mouse Mutant Archive [EMMA]), the gene trap cassette that it carries is inserted roughly 1 kb downstream from the insertion site of the gene trap cassette present in *Tpcn1*^XG716^. On the basis of results from our present study, it is possible that the line *Tpcn1*^LEXKO-471^ still sustains the initiation of transcription just upstream from exon 3, giving rise to *Tpcn1B*. Additionally, posttranscriptional skipping of the splice acceptor sequence present in the gene trap cassette could be occurring in the *Tpcn1*^LEXKO-471^ line, resulting in *Tpcn1A*. Based on our findings presented here, we believe that it will be important to confirm the absence of *Tpcn1* expression in this line, although data on this were not presented in that study (42), in order to confirm its proposed status as *Tpcn1*^−/−^. The other two studies (28, 29) used animals classified as *Tpcn1/2* double knockouts with targeted disruption of the *Tpcn1* gene by deletion of unspecified sequences from the 5′ UTR to the intron between exons 2 and 3 via a LoxP/Cre strategy, but again, the authors provided no data demonstrating the *Tpcn1* expression status of this mouse line. As before, such a deletion may potentially result in the production of a *Tpcn1B* transcript. We suggest that in this case also it will be important to verify that there is the absence of *Tpcn1* expression in order to confirm the *Tpcn1*^−/−^ status.

### Expression of *Tpcn1A* and *Tpcn1B* is abolished in *Tpcn1*^T159^ mice.

**FIG 3:**
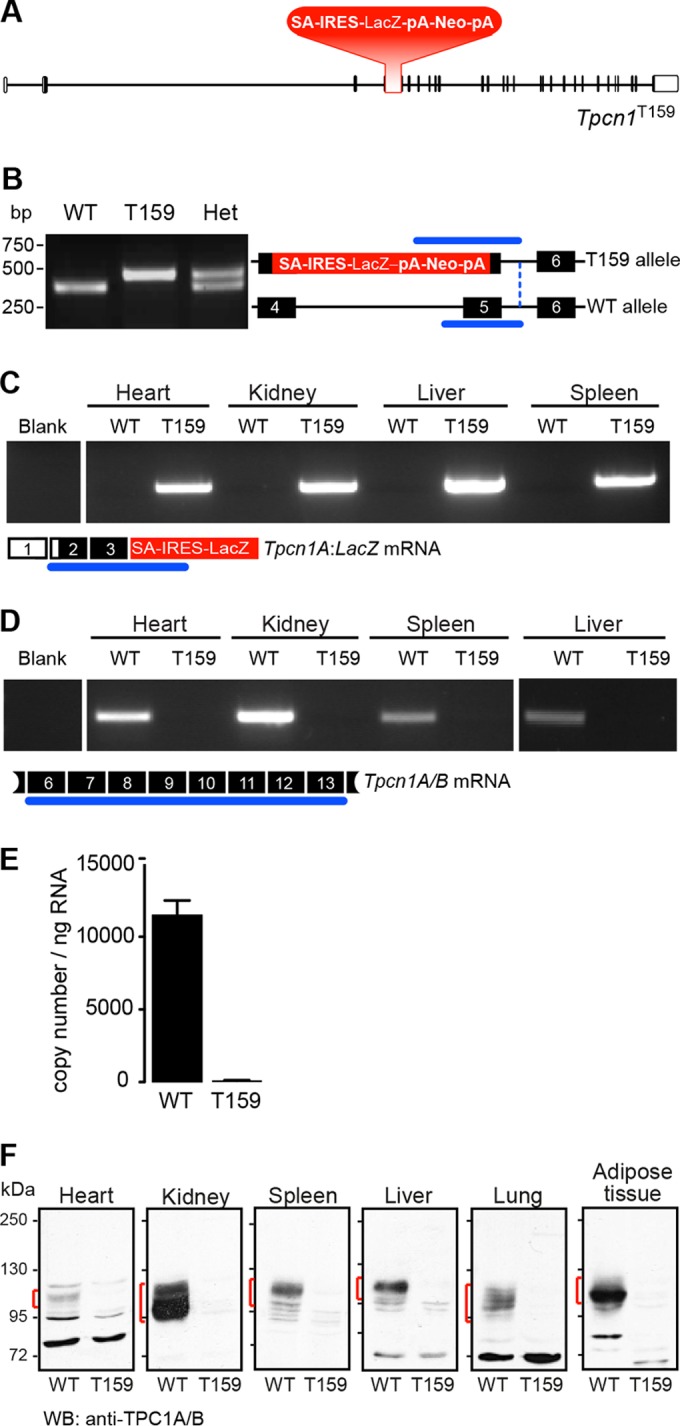
A targeted mutation in the *Tpcn1*^T159^ gene abolishes *Tpcn1* expression. (A) Schematic representation of *Tpcn1*^T159^ gene structure based on the gene with ID no. 252972 and the cDNA sequence with GenBank accession number NM_145853.2. Red blocks, knockout cassette (SA, splice acceptor; IRES, internal ribosomal entry site; pA, polyadenylation signal). The neomycin resistance gene (Neo) is under the control of a promoter present downstream of the LacZ gene. Vertical segments, exons; unfilled boxes, UTRs. (B) Genotyping of mice for WT and *Tpcn1*^T159^ alleles. Numbered black blocks, coding exons; blue lines, PCR-amplified regions. (C, D) RT-PCR results for expression of *Tpcn1* transcripts in tissues from WT or *Tpcn1*^T159^ homozygote mice. Blank, reactions with no RNA; numbered white boxes, noncoding exons. (C) Expression of the truncated chimeric *Tpcn1A-LacZ* transcript from the *Tpcn1*^T159^ allele. (D) Expression of the *Tpcn1A/B* transcript determined by probing for regions downstream from the gene targeted disruption. (E) Copy number of *Tpcn1A/B* transcripts detected in liver from WT and homozygote *Tpcn1*^T159^ animals using RT-qPCR. Bars correspond to the mean ± SEM for 4 animals. (F) Immunoblotting analysis of TPC1A/B present in membrane samples from tissues of WT or *Tpcn1*^T159^ homozygote mice using an anti-TPC1 antibody recognizing the C-terminal region of TPC1 proteins. Red markings, immunoreactive bands not present in *Tpcn1*^T159^ samples.

In order to obtain mice with a total lack of expression of both *Tpcn1A* and *Tpcn1B* transcripts, we investigated a transgenic *Tpcn1* line with disruption of exons common to both transcripts. This *Tpcn1* line, *Tpcn1*^T159^, carries a targeted knockout cassette replacing sequences corresponding to exons 4 and 5 (Fig. 3A). Mice were genotyped using a three-primer PCR protocol (Fig. 3B), and *Tpcn1A/B* expression was assessed by RT-PCR. Predictably, tissues from homozygote *Tpcn1*^T159^ mice, but not those from WT mice, expressed chimeric mRNAs comprising *Tpcn1* exons upstream from the targeted site joined to *LacZ* sequences present within the knockout cassette (Fig. 3C). Similar to the findings for *Tpcn1*^XG716^ mice, we suggest that expression of β-galactosidase from *Tpcn1*^T159^ animals can be used as a reporter for promoter activity. Moreover, due to the location of the reporter cassette in this transgenic line, we predict that expression of β-galactosidase may originate from the two promoters that give rise to *Tpcn1A* and *Tpcn1B*, whereas in *Tpcn1*^XG716^ mice, expression of β-galactosidase is driven solely by the promoter responsible for expression of *Tpcn1A*; *w*e suggest that this could be exploited as a tool to investigate different regulatory traits of the two promoters.

In order to evaluate the effects of the targeted disruption of exons 4 and 5 on both *Tpcn1A* and *Tpcn1B* expression, RT-PCR analysis targeting sequences that are downstream from exon 5 and that are therefore shared by both forms of *Tpcn1* was performed. Our results indicated that while *Tpcn1* was expressed in tissues from WT mice, *Tpcn1*^T159^ homozygotes had no detectable *Tpcn1A/B* expression (Fig. 3D). Thus, RT-qPCR analysis using liver RNA from *Tpcn1*^XG716^ homozygotes confirmed that *Tpcn1* mRNA expression was essentially absent in *Tpcn1*^T159^ animals (Fig. 3E). Moreover, using anti-TPC1 antibodies targeting the C terminus of the TPC1 protein, the respective endogenous TPC1 protein(s) could be readily detected by immunoblot analysis in membrane preparations from WT mouse tissues but not from equivalent samples obtained from *Tpcn1*^T159^ homozygote animals (Fig. 3F). We also observed tissue-specific differences in the apparent molecular masses of immunoreactive bands, which could be due to differences in TPC1A/TPC1B protein ratios and/or differences in glycosylation levels, a posttranslational modification previously seen in the mouse TPC1 protein (11). We conclude that homozygote *Tpcn1*^T159^ mice have fully knocked out expression of both *Tpcn1* isoforms, and these mice are referred to here as *Tpcn1*^−/−^ mice. A recent report of a study using transgenic animals with the disruption of exon 3 of *Tpcn1* showed a lack of TPC1 proteins in testicular tissue and sperm (43), further demonstrating that targeting of a common exon between *Tpcn1A* and *Tpcn1B* results in a demonstrable *Tpcn1*^−/−^ status.

### Localization of mouse TPC1B protein.

**FIG 4:**
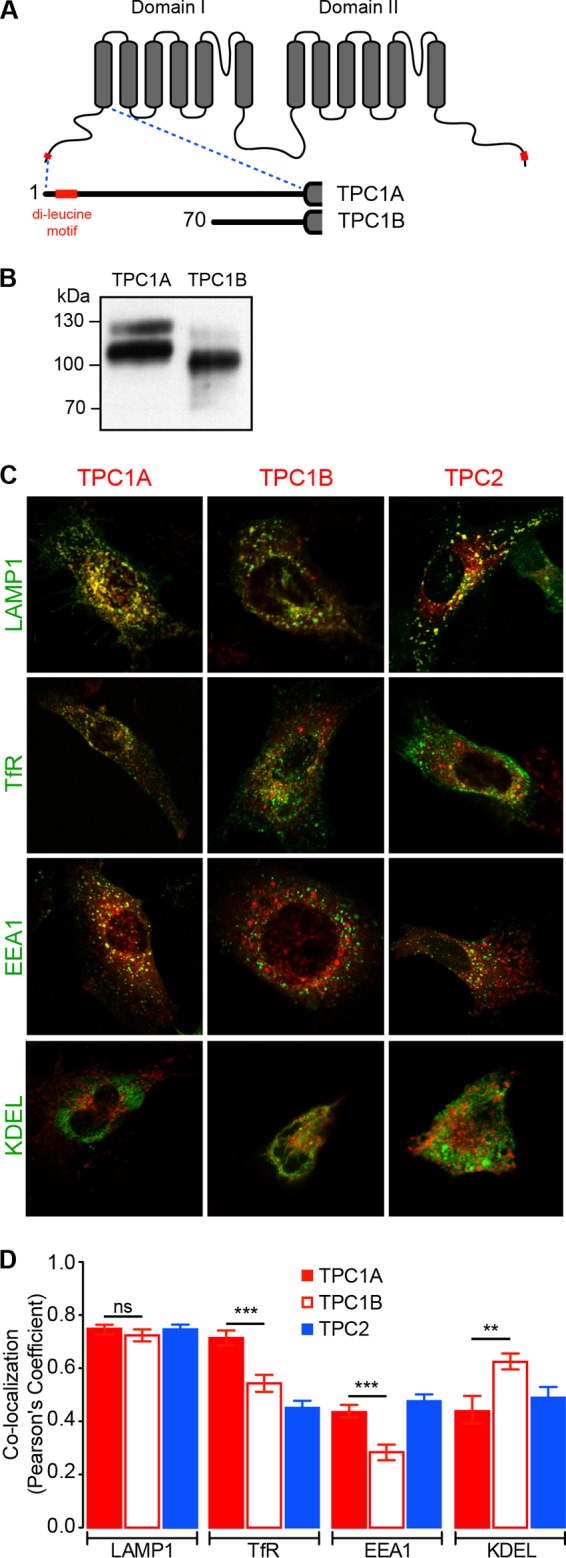
Colocalization of mouse TPC1A, TPC1B, and TPC2 with distinct endo-lysosomal organelle markers. (A) Schematic representation of the transmembrane segments (gray boxes) of TPC1 and the N-terminal truncated TPC1B isoform predicted to be produced from the *Tpcn1B* transcript. Red, the dileucine motif(s) present in TPC1A and TPC1B. The numbers correspond to the starting amino acid residue. (B) Immunoblotting analyses of expression of mCherry-tagged TPC1A and TPC1B using an antimultired antibody. (C, D) Representative images of immortalized MEFs coexpressing GFP-tagged organelle markers (Lamp1 for late endosomes/lysosomes, TfR for recycling endosomes, EEA1 for early endosomes, and KDEL for ER) and mCherry-tagged TPC1A, TPC1B, and TPC2. (D) Compilation of colocalization coefficient between organelle markers and TPC proteins. Results are represented as the mean ± SEM for 10 to 32 cells. ns, no significant difference (*P* > 0.05); **, *P* < 0.01; ***, *P* < 0.001.

TPC proteins from several different species have been shown to be glycosylated and to localize within organelles of the endo-lysosomal system, with TPC2 being restricted to late endosomes/lysosomes and TPC1A showing a broader pattern of localization (10–12, 16). Sorting of proteins within the endo-lysosomal system is often determined by small amino acid motifs present within their sequences (17), and deletion of such a dileucine motif in human TPC2 causes mistargeting of the resultant protein to the plasma membrane (18). Importantly, translation of the *Tpcn1B* transcript is predicted to result in a protein, TPC1B, with an N-terminal truncation of the first 69 amino acid residues of TPC1A, thereby removing one of its two dileucine motifs (Fig. 4A). We therefore examined what effect such a truncation has upon expression and localization of the resulting TPC1B protein.

Heterologous expression of mouse TPC1B tagged with a C-terminal mCherry sequence results in a protein with a molecular mass lower than that of the equivalent TPC1A, as expected, and a reduced level of the fully glycosylated form compared to the level in TPC1A (Fig. 4B); whether this difference is due to changes in protein folding and maturation (44) is unknown.

We next examined the intracellular localization assessed by determination of the colocalization of TPC1A, TPC1B, and TPC2 with GFP-tagged organelle marker proteins in mouse embryonic fibroblasts (MEFs) (Fig. 4C). While TPC1A was found to colocalize to the same extent with markers for late endosomes/lysosomes (Lamp1) and recycling endosomes (TfR) (Fig. 4D), TPC1B localized mainly to late endosomes/lysosomes and had decreased localization within early and recycling endosomes compared to TPC1A (Fig. 4D). Localization of mouse TPC2 also seemed to occur preferentially in late endosomes/lysosomes (Fig. 4D), as previously described for other species (10, 16). This observation would appear to indicate that the dileucine motif present at the N-terminal tail of TPC1A is not the sole determinant for endo-lysosomal localization (a similar observation has been reported for human TPC1A [18]), with other targeting motifs, including possible tyrosine-based sorting signals (17) and the C-terminal tail dileucine motif (Fig. 4A), having a contributory role. However, the N-terminal dileucine motif, in conjunction with other targeting signals, may play a role in sorting of TPC1A into specific endo-lysosomal organelles.

Previously, we have identified heterodimer formation between TPC1 and TPC2 (45). Given the different patterns of subcellular localization of TPC1A and TPC1B that we have identified in this study, with the latter showing a localization more similar to that of TPC2, an interesting question for future study would be to investigate whether TPC2 is more likely to interact with this isoform than with TPC1A in the *in vivo* cellular environment, since this might have functional consequences.

### Mouse embryonic fibroblasts derived from *Tpcn1*^−/−^ or *Tpcn2*^−/−^ mice show normal pH and endo-lysosomal morphology.

**FIG 5:**
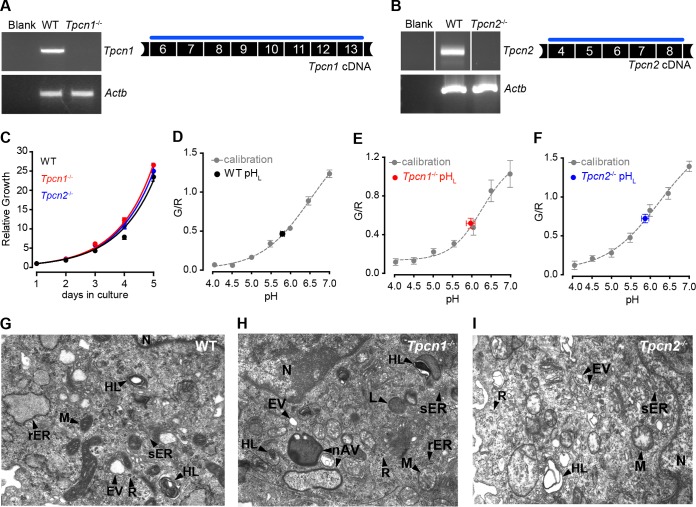
Growth properties, endo-lysosomal pH, and morphology of MEFs from *Tpcn1*^−/−^ and *Tpcn2*^−/−^ embryos. (A, B) RT-PCR analysis of *Tpcn1* (A) and *Tpcn2* (B) expression in MEFs derived from WT or *Tpcn1*^−/−^ and *Tpcn2*^−/−^ embryos. Amplified cDNA regions correspond to the numbered exonic sequences covered by the blue line. Expression of *Actb* was used as a control. (C) Growth curves of primary MEFs in culture. (D to F) Determination of endo-lysosomal luminal pH (pH_L_) in MEFs from WT (D), *Tpcn1*^−/−^ (E), and *Tpcn2*^−/−^ (F) embryos. Values were determined against a calibration curve of the ratios of fluorescein/Texas Red fluorescence (*G*/*R*) against a series of defined pH values in fluorescent dextran-loaded MEFs of each genotype. (G to I) Organellar morphology assessed by electron microscopy of MEFs from WT (G), *Tpcn1*^−/−^ (H), and *Tpcn2*^−/−^ (I) embryos. Primary MEFs (passage numbers, <5) were used in all experiments. nAV, nascent autophagic vesicle; rER, rough endoplasmic reticulum, sometimes showing a dilated appearance; sER, smooth endoplasmic reticulum, sometimes showing a granular appearance; EV, endocytic vesicles; HL, heterogeneous lysosomes, including multilamellar bodies and multivesicular structures; L, lysosomes; M, mitochondria; N, nucleus; R, free ribosomes.

Low-passage-number MEFs (<5 passages) prepared from WT and *Tpcn1*^−/−^ or *Tpcn2*^−/−^ embryos were used as a system to analyze the impact of the knocked out expression of TPC1A/B or TPC2 on cellular functions. RT-PCR analysis of *Tpcn1A/B* and *Tpcn2* expression in these cells revealed that while WT MEFs contained readily detectable levels of *Tpcn1A/B* and *Tpcn2* mRNAs, no expression could be observed for *Tpcn1A/B* or *Tpcn2* in MEFs from the respective knockout embryos (Fig. 5A and B). Results from growth rate experiments showed that ablation of TPC1A/B or TPC2 expression did not significantly affect the growth of MEFs, with doubling times being 0.89 days (95% confidence interval [CI], 0.87 to 0.91 days) for the WT cells, 0.84 days (95% CI, 0.84 to 0.85 days) for *Tpcn1*^−/−^ cells, and 0.86 days (95% CI, 0.84 to 0.88 days) for *Tpcn2*^−/−^ cells (Fig. 5C).

Acidic luminal pH is integral to the functions of the endo-lysosomal system, with ion fluxes contributing to the maintenance of the luminal pH (6, 46). We therefore assessed the impact of TPC loss upon endo-lysosomal pH using a ratiometric fluorescence assay. In line with what we have previously reported for HEK293T cells overexpressing sea urchin TPC proteins (16), no significant differences in the luminal pH were observed between the different genotypes analyzed, with pH values being 5.82 ± 0.06 for WT MEFs, 5.89 ± 0.12 for *Tpcn1*^−/−^ MEFs, and 5.87 ± 0.10 for *Tpcn2*^−/−^ MEFs (Fig. 5D to F). It was also recently reported that overexpression of TPC2 in HeLa cells causes only a modest increase (0.3 unit) of the luminal pH, with no major impact on lysosomal functions dependent on acidic luminal pH being detected (31). Overall, these combined results suggest that manipulation of TPC expression does not majorly affect the resting acidic luminal pH.

Previously, it was shown that overexpression of human TPC2 in cell lines induces a lysosomal storage disease phenotype (16) and an increased accumulation of autophagosomes (31, 34), possibly due to an inhibition of autophagosomal/lysosomal fusion (31). However, our results from electron microscopy revealed that knocked out expression of TPC1A/B or TPC2 had no major impact on the ultrastructure of MEFs (Fig. 5G to I), with cells showing a complex organelle population characteristic of this cell type (47–49), irrespective of genotype, although we cannot exclude the possibility that the effects of the loss of TPC1A/B or TPC2 become apparent only under stress conditions. Such a possibility is supported by a previous report showing that the downregulation of TPC2 in HEK293T cells (either by a dominant negative TPC2 mutant or by the NAADP antagonist Ned-19) impaired the accumulation of autophagosomes induced by the expression of leucine-rich repeat kinase 2 but not under control conditions (34).

### Knockout expression of *Tpcn1A/B* impairs cholera toxin trafficking.

**FIG 6:**
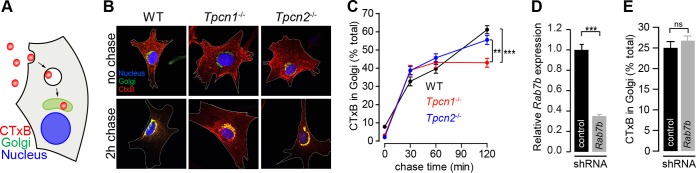
Impact of knockout expression of *Tpcn1A/B* or *Tpcn2* in cholera toxin trafficking. (A) Schematic representation of CTxB trafficking from the plasma membrane to the Golgi apparatus. (B) Representative images for 0-min (no chase) and 120-min chase periods at 37°C, after 30 min incubation on ice with Alexa Fluor 555-labeled CTxB. Cells were immunolabeled with an antibody against the Golgi apparatus marker protein GM130. Cell boundaries are identified with a white broken line. (C) Quantification of CTxB levels in the Golgi apparatus at the indicated chase time after the CTxB binding period (*n* = 56 to 93). (D, E) Effects of treatment of WT MEFs with shRNA (shRNA targeting *Rab7b* or a scrambled sequence control) on relative *Rab7b* mRNA levels determined by RT-qPCR (D) and on CTxB levels in the Golgi apparatus after a chase period of 120 min (E) (*n* = 32 to 36). Data points correspond to the mean ± SEM. ns, no significant difference (*P* > 0.05); **, *P* < 0.01; ***, *P* < 0.001. Primary MEFs (passage numbers, <5) were used in all experiments.

Cholera toxin retrograde transport from the plasma membrane to the endoplasmic reticulum via the Golgi apparatus occurs through early and recycling endosomes (50) (Fig. 6A), and we have previously shown that abnormally elevated TPC1 or TPC2 expression in HEK293T cells results in an endo-lysosomal blockage of subunit B of cholera toxin (CTxB) (16). We therefore assessed the impact of knocked out expression of TPC1A/B or TPC2 in this pathway by measuring the kinetics of fluorescently labeled CTxB accumulation in the Golgi apparatus by colocalization with GM130, a Golgi apparatus marker protein (Fig. 6B and C). In WT cells, the amount of CTxB in the Golgi apparatus increased in a time-dependent manner, reaching 61.2% ± 2.2% of the total amount of CTxB in the cell by a chase period of 2 h (Fig. 6C). Although no significant differences were observed for *Tpcn2*^−/−^ MEFs (55.5% ± 2.4%), *Tpcn1*^−/−^ MEFs showed a significant reduction in the maximum level of CTxB accumulating in the Golgi apparatus, reaching a lower plateau of 43.0% ± 2.4% after a chase time of 30 min (Fig. 6C), corresponding to a 30% reduction compared to the level of accumulation in WT cells. This impairment became more apparent compared to the results obtained in MEFs derived from embryos lacking the critical vesicular protein sorting factor Vps54 (50% reduction [51]), whose single amino acid mutation is responsible for the wobbler mouse phenotype (52).

Our results suggest that a lack of TPC1A/B has a significant impact in this retrograde transport pathway, in line with our previous results showing that overexpression of TPC1 has a more dramatic effect on CTxB trafficking than overexpression of TPC2 (16). The different contributions of TPC1-regulated versus TPC2-regulated pathways in CTxB retrograde transport can be explained by the distinct localization patterns of TPC1 and TPC2; transport of CTxB from the plasma membrane to the ER (via the Golgi apparatus) has been shown to occur through early and recycling endosomes, circumventing the late endosome pathway (50), and while TPC1A colocalizes strongly with markers for recycling endosomes (Fig. 4) (10), TPC2 is predominantly localized to late endosomes/lysosomes (Fig. 4) (10, 12). This explanation is further substantiated by our results obtained in MEFs with disrupted expression of Rab7b (a member of the Rab GTPase protein family, whose members are recognized to be master regulators of intracellular trafficking events [53]), which, like TPC2, is a late endosome/lysosome-associated protein (54, 55); while in cells treated with shRNA targeting *Rab7b* (*Rab7b*-shRNA) the level of *Rab7b* mRNA was reduced to 35.1% ± 1.5% of the level in control shRNA-treated cells (Fig. 6D), no significant effect on the proportion of CTxB in the Golgi apparatus was observed after a chase period of 2 h (*Rab7b*-shRNA-treated cells, 25.0% ± 1.5% of the total amount of CTxB; control shRNA-treated cells, 26.7% ± 1.2% of the total amount of CTxB) (Fig. 6E). This contrasts with the previously reported effect of *Rab7b* depletion in HeLa cells on CTxB accumulation in the Golgi apparatus (55), where Rab7b has been shown to partially colocalize with *trans*-Golgi network and Golgi apparatus markers, in addition to late endome/lysosome markers (55). However, we cannot dismiss the possibility that the lack of an effect of *Rab7b* depletion in MEFs is due to a diminished prevalence of the Rab7b-mediated pathway in this cell type or that the extent of reduction of *Rab7b* expression achieved by shRNA is not sufficient to unmask its role in this retrograde transport pathway in MEFs.

On the basis of results shown here, it is likely that the TPC1 (and, in particular, TPC1A) function might be important not only in the pathogenesis caused by CTxB but also in the pathogenesis caused by other bacterial toxins that use a similar trafficking pathway (56).

### Knockout expression of *Tpcn2* delays ligand-induced PDGFRβ degradation.

**FIG 7:**
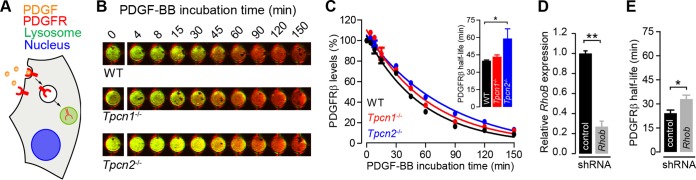
Impact of knockout expression of *Tpcn1A/B* or *Tpcn2* in PDGFRβ degradation. (A) Schematic representation of PDGFR internalization from the plasma membrane to the lysosomes and degradation induced by the ligand PDGF. (B) Representative images of MEFs treated with 20 ng/ml PDGF-BB (in the presence of 20 μg/ml cycloheximide) for the indicated times at 37°C. Green and red signals, PDGFRβ and CellTag 700 staining, respectively; yellow, high levels of PDGFRβ. (C) The levels of PDGFRβ detected with an anti-PDGFRβ antibody were normalized to the number of cells detected with CellTag 700 stain. Data points were fitted to an exponential decay curve, and half-lives were determined for each genotype (in the graph, *n* = 3). (D, E) Effects of treatment of WT MEFs with shRNA (shRNA targeting *Rhob* or a scrambled sequence control) on relative *Rhob* mRNA levels determined by RT-qPCR (D) and on the half-life of PDGFRβ in PDGF-BB-treated cells (E) (*n* = 3). Data points correspond to the mean ± SEM. *, *P* < 0.05; **, *P* < 0.01. Primary MEFs (passage numbers, <5) were used in all experiments.

As TPC proteins localize to late endosomes/lysosomes, we next looked at what impact knocked out expression of *Tpcn1A/B* or *Tpcn2* has on another pathway relying on trafficking, this time from the plasma membrane to lysosomes. Binding of ligands to their receptor tyrosine kinase (RTK) targets at the plasma membrane elicits a signaling cascade that results in receptor internalization and transport to lysosomes, culminating in receptor degradation (57) (Fig. 7A), and the kinetics of receptor degradation have been widely studied to assess defects in this pathway, with epidermal growth factor receptor (EGFR) degradation often being chosen as the prototypic model system.

In this study, we opted to study the platelet-derived growth factor receptor β (PDGFRβ), as it is highly expressed in fibroblasts, allowing us to study its endogenous levels instead of relying on heterologous expression, which can cause artifacts due to pathway saturation. We therefore examined the rate of degradation of endogenous platelet-derived growth factor receptor β (PDGFRβ) induced by addition of the ligand PDGF-BB by quantifying the levels of the endogenous protein detected by immunofluorescence using an anti-PDGFRβ antibody (Fig. 7B). In WT MEFs, the half-life of PDGFRβ in the presence of PDGF-BB was 39.9 ± 0.6 min, and no significant difference from that in *Tpcn1*^−/−^ MEFs was observed (43.3 ± 1.6 min) (Fig. 7C). However, in *Tpcn2*^−/−^ MEFs, the half-life of PDGFRβ in the presence of PDGF-BB was significantly extended to 59.1 ± 8.4 min, an approximately 50% increase relative to that in WT MEFs (Fig. 7C). The significance of this increased half-life is particularly evident compared to that observed in cells treated with shRNA targeting *Rhob* (*Rhob*-shRNA; *Rhob* is a small GTPase previously shown to regulate PDGFRβ trafficking and signaling in vascular smooth muscle cells [58]); *Rhob*-shRNA treatment of WT MEFs resulted in a reduction in the level of *Rhob* expression to 27% ± 5.5% relative to that in control shRNA-treated cells (Fig. 7D), with a concomitant 36% increase in the PDGFRβ half-life relative to that in scrambled shRNA-treated WT MEFs being detected (Fig. 7E).

The lower rate of degradation of PDGFRβ in *Tpcn2*^−/−^ cells is likely due to differences in trafficking of the activated receptor from the plasma membrane to lysosomes, rather than differences in receptor degradation by lysosomal cathepsins (whose activity is dependent on acidic pH), as no differences in luminal pH were observed among the different genotypes (Fig. 5D to F).

In summary, our study of endo-lysosomal trafficking in *Tpcn1*^−/−^ and *Tpcn2*^−/−^ MEFs indicates that the two TPC isoforms have important but distinct roles in endo-lysosomal trafficking. Moreover, our findings suggest that defects in TPC expression and function might underlie some disease states caused by impaired endo-lysosomal trafficking. While our results confirm the importance of TPC1 and TPC2 function in endo-lysosomal pathways, they also highlight important differences in the role of TPC1 versus that of TPC2. Whether this is due to differences in the biological activities of the two channel proteins and/or a different organellular pattern of localization (as discussed above) remains to be determined; it is likely, however, that both aspects might contribute to the differences observed. In regard to their biological activity, TPCs have been shown to behave as NAADP-regulated Ca^2+^-permeant channels (29), and the release of Ca^2+^ from endo-lysosomal organelles is necessary for different aspects of endo-lysosomal function, including organelle fusion (6). Additionally, evidence that TPCs also have the capacity to act as Na^+^ channels was recently provided (29), and although the effects of Na^+^ release from endo-lysosomes on organelle fusion have not been studied, it is likely that changes in membrane potential as a result of Na^+^ release will have an impact. In either scenario, a lack of TPC1A/B or TPC2 proteins is likely to have an impact on the fusogenic potential of this pathway.

## Supplementary Material

Supplemental material
