# Supplementary material for: TPC1 Has Two Variant Isoforms, and Their Removal Has Different Effects on Endo-Lysosomal Functions Compared to Loss of TPC2
Source: Mol Cell Biol. 2014 Nov;34(21):3981–92. doi: 10.1128/MCB.00113-14 (PMC4386455; doi:10.1128/MCB.00113-14)
Supplement: Supplemental material [file supp_34_21_3981__index.html]

TPC1 Has Two Variant Isoforms, and Their Removal Has Different Effects on Endo-Lysosomal Functions Compared to Loss of TPC2 — Supplemental material 

# TPC1 Has Two Variant Isoforms, and Their Removal Has Different Effects on Endo-Lysosomal Functions Compared to Loss of TPC2

## Supplemental material

**Files in this Data Supplement:**

- Supplemental file 1 -

  Tables S1 and S2 (Primers and PCR parameters for determination of gene trap insertion site [S1] and genotyping mice [S2]), S3 (Primers and PCR annealing temperatures), and S4 (Primers and probes for qPCR)

  PDF, 47K
