# Supplementary material for: TPC1 Has Two Variant Isoforms, and Their Removal Has Different Effects on Endo-Lysosomal Functions Compared to Loss of TPC2
Source: Mol Cell Biol. 2014 Nov;34(21):3981–92. doi: 10.1128/MCB.00113-14 (PMC4386455; doi:10.1128/MCB.00113-14)
Supplement: Supplemental material [file MCB.00113-14_zmb999100621so1.pdf]

TPC1 has two variant isoforms and their removal has different effects on endo-lysosomal functions compared to loss of TPC2

Margarida Ruas, Kai-Ting Chuang, Lianne C Davis, Areej Al-Douri, Patricia W Tynan, Ruth Tunn, Lydia Teboul, Antony Galione, John Parrington

Table S1. Primers and PCR parameters used for determination of the gene trap insertion site in the *Tpcn1*^{XG716} allele.

<table>
<tr><th>Primers</th><th>PCR parameters</th></tr>
<tr><td>F1: GCAGAGCGAGGTATGTAGGC
R1: TTGCCGCCTCTTGATAATTC</td><td rowspan="4">92 ºC (2 min)

92 ºC (10 s)
56 ºC (15 s)
68 ºC (6 min)
} 10 x

92 ºC (10 s)
56 ºC (15 s)
68 ºC (6 min + 20s/cycle)
} 20 x

72 ºC (6 min)</td></tr>
<tr><td>F1: GCAGAGCGAGGTATGTAGGC
R2: GGCAGATCTTTGAAGGCAAG</td></tr>
<tr><td>F1: GCAGAGCGAGGTATGTAGGC
R3: ATGATGAGGCTCTTGGGATG</td></tr>
<tr><td>F2: TGACGGTGAAAACCTCTGACAC
R4: GGCAGATCTTTGAAGGCAAG</td></tr>
</table>

F (forward), R (reverse).

Table S2. Primers and PCR parameters used for genotyping mice.

<table>
<tr><th>Mouse line</th><th>Primers</th><th>Amplicon sizes (bp)</th><th>PCR parameters</th></tr>
<tr><td>Tpcn1^{XG716}</td><td>I2 F: AACTTGTTAGGATCTCCCCGC
I2 R: GGCAGATCTTTGAAGGCAAG
βgeo F: TGACGGTGAAAACCTCTGACAC</td><td>WT allele: 496
XG716 allele: 383</td><td rowspan="2">94 ºC (5 min)

94 ºC (15 s)
56 ºC (30 s)
72 ºC (1 min)
} 30 x

72 ºC (6 min)</td></tr>
<tr><td>Tpcn1^{T159}</td><td>I4 F: CTGGCATCTTGAGGTTTGGT
I5 R: GGGCTACACTCCCAAGCATA
βgal F: CCAGCTCATTCCTCCCACTC</td><td>WT allele: 376
T159 allele: 459</td></tr>
</table>

Primers for genotyping *Tpcn1*^{XG716} were designed based on results from Figure 1C.
Primers for genotyping *Tpcn1*^{T159} were designed based on information available at EMMA.
I (intron), E (exon), βgeo (βgal/neo cassette).

Table S3. Primers and PCR annealing temperatures used for studying expression of transcripts.

Transcript	Primers	Amplicon sizes (bp)	Anneling temperature
Tpcn1A	E2 F: AGTTTAGATGACGATGTGCCG<br>E9 R: TTGACGATGCTGTTCTCCAG	819	58
Tpcn1B	I2 F: CCACCACGGCTTCTGAGTT<br>E6 R: CACAATGGCCTCGATGAAC	497	57
Tpcn1B	I2 F: CCACCACGGCTTCTGAGTT<br>E27 R: AGAAGAGGCTGGCTTGACG	2408	58
Tpcn1A/B	E4 F: ACCTCTTCGTCCACAACCAC<br>E9 R: TTGACGATGCTGTTCTCCAG	516	58
Tpcn1A/B	E6 F: ATTTTCCTGGTGGACTGTCG<br>E13 R: CAGAGCAGCGACTTCGTAAA	606	57
Tpcn1A:βgeo	E2 F: AGTTTAGATGACGATGTGCCG<br>βgeo R: GACAGTATCGGCCTCAGGAAGATCG	438<br>(in XG716)	58
		1335<br>(in T159)	58
Actb	E2 F: GATGACGATATCGCTGCGCTGGTCG<br>E4 R: GCCTGTGGTACGACCAGAGGCATACAG	447	58

I (intron), E (exon), βgeo (βgal/neo cassette).

Table S4. Primers and probes used for qPCR

Transcript	Primers	Universal Probe
Tpcn1A/B	F: TCCAAGGCCTTCCAGTATTTC<br>R: CTCCACCAGGATCCAGACAC	77
Gapdh	F: AGCTTGTCATCAACGGGAAG<br>R: TTTGATGTTAGTGGGGTCTCG	9
Cyc1	F: ACCTGGTGGGAGTGTGCTAC<br>R: CATCATCATTAGGGCCATCC	10
Actb	F: CTAAGGCCAACCGTGAAAAG<br>R: ACCAGAGGCATACAGGGACA	64
Rn18s	F: CTCAACACGGGAAACCTCAC<br>R: CGCTCCACCAACTAAGAACG	77
Rhob	F: TATGTGGCGGACATCGAG<br>R: AGAGCGGCCGTAAACGAT	79
Rab7b	F: TCGAGGAATACCAGACCACA<br>R: GCTTCAAAGTTGTGTCATCCAA	55

Primer pairs and their matching Universal ProbeLibrary probes were designed by Roche Universal ProbeLibrary Assay Design Centre.
